# Supplementary figures and images for: Questioning the role of selected somatic PIK3C2B mutations in squamous non-small cell lung cancer oncogenesis
Source: PLoS One. 2017 Oct 31;12(10):e0187308. doi: 10.1371/journal.pone.0187308 (PMC5663493; doi:10.1371/journal.pone.0187308)

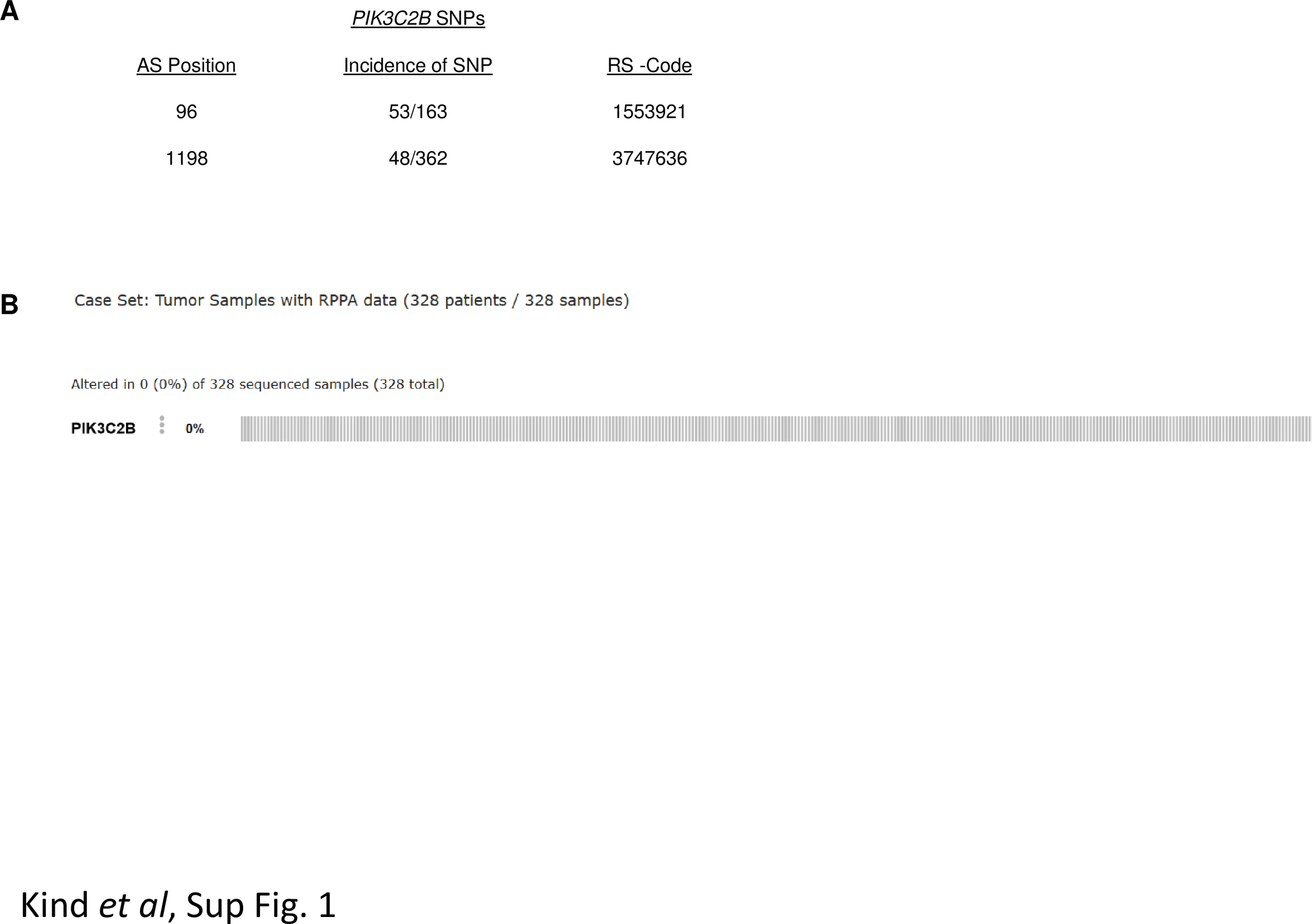

Supplement: S1 Fig — A Alterations in PI3KC2β protein expression measured with reverse-phase protein array (RPPA). Z-score threshold ±1 B Table with incidence rate and position of found PIK3C2B single nucleotide polymorphisms (SNP). (TIF) [file pone.0187308.s001.tif]

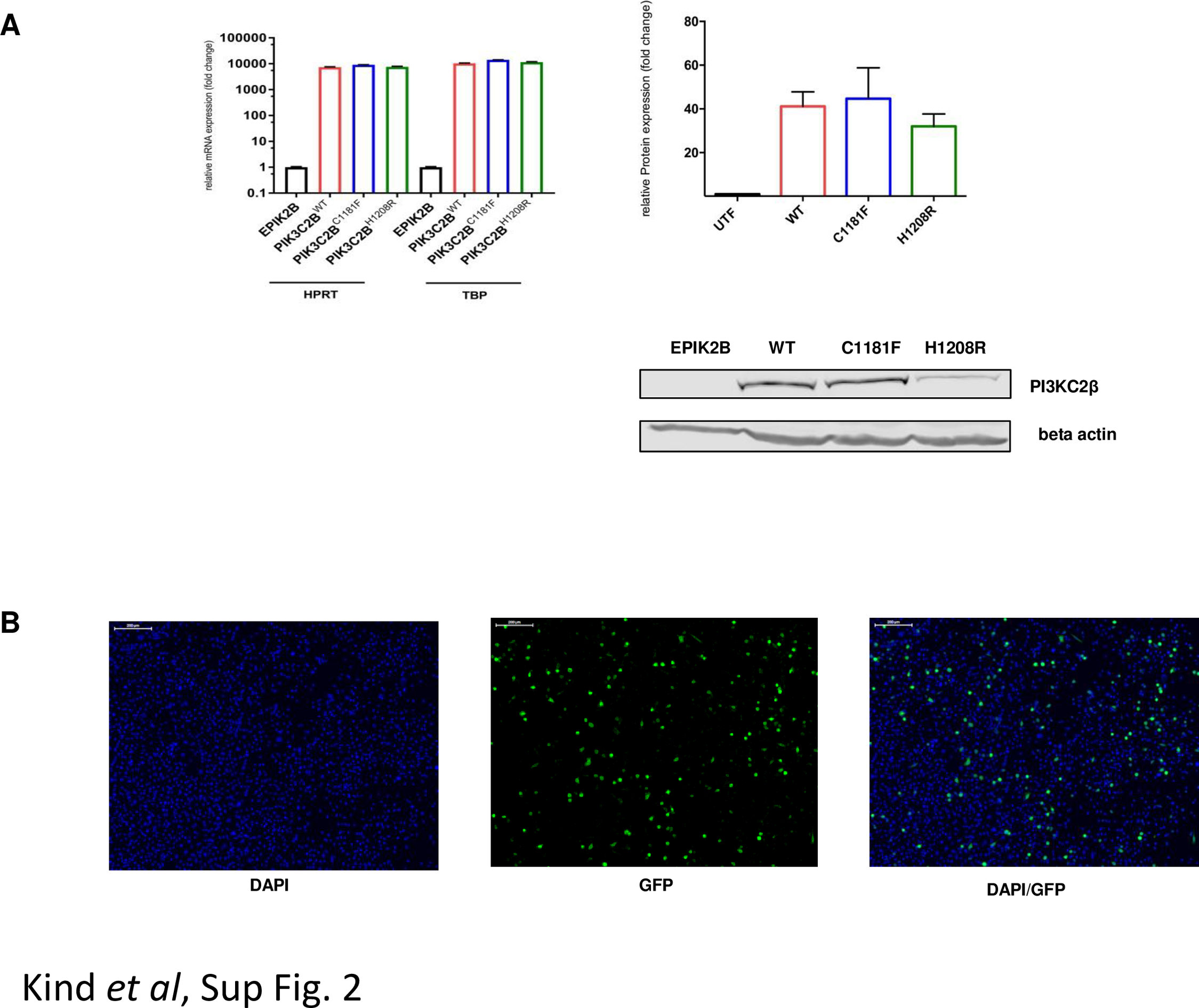

Supplement: S2 Fig — A Relative mRNA expression of PIK3C2B after transfection of PC9 cells (36h) with plasmid constructs. PIK3C2B expression was normalized to housekeeping genes hypoxanthin phosphoribosyltransferase 1 (HPRT) and TATA box binding protein (TBP). Means ± SEM; n = 2 independent experiments. Relative protein expression of PIK3C2β after transfection of PC9 cells (36h) with plasmid constructs. Mean ± SEM; n = 2 independent experiments B Transfection efficacy measured in PC9 cells via immunofluorescence. Cells were transfected with a GFP plasmid for 36h. Staining with DAPI (blue). Share of GFP+ cells: 10–20%. (TIF) [file pone.0187308.s002.tif]
